# Improved decisions for unknown behaviours in interactive dynamic influence diagrams

**DOI:** 10.1007/s10462-025-11355-y

**Published:** 2025-08-30

**Authors:** Yinghui Pan, Mengen Zhou, Biyang Ma, Yifeng Zeng, Yew-soon Ong, Guoquan Liu

**Affiliations:** 1 School of Artificial Intelligence, Shenzhen University, Shenzhen, China; 2 School of Computer Science & Research Institute of Embodied Interaction Science and Technology, Minnan Normal University, Zhangzhou, China; 3 School of Computer Science, Northumbria University, Newcastle, UK; 4 School of Computer Science and Engineering, Nanyang Technological University, Singapore, Singapore; 5 Institute of FinTech, Fudan University, Shanghai, China

**Keywords:** Dynamic response optimization, Multiagent systems, Evolutionary computation

## Abstract

Interactive dynamic influence diagrams (I-DIDs) are a general decision framework for a subject agent who interacts with other agents (of either collaborative or competitive) in a common environment with partial observability. The subject agent aims to optimize its decision-making (response strategy) while other agents concurrently adapt their behaviors over time. The I-DID model has faced a long-term challenge when other agents exhibit unknown behaviors that go beyond what the subject agent has planned for prior to their interactions. This is because the subject agent does not hold the capability of modeling unknown behaviours of other agents in traditional I-DID techniques. In this article, we adapt two different swarm intelligence (SI) techniques to develop new behaviours for other agents in I-DIDs. The SI-based algorithms have the strength of generating a collective set of behaviours that could potentially contain various types of agents’ behaviours. We theoretically analyze how the two algorithms impact the subject agent’s decision quality, and empirically demonstrate the algorithm performance in two commonly used problem domains.

**Supplementary Information:**

The online version contains supplementary material available at 10.1007/s10462-025-11355-y.

## Introduction

Interactions between agents generally arise when multiple autonomous agents operate in a common environment under uncertainty. An agent might make a change to some feature of the environment that in turn impacts other agents who may possibly act in a similar way. A subject agent needs to optimize its response strategy given its beliefs about both the environmental states and the other agents’ behaviours. The response becomes difficult when other agents exhibit unexpected behaviours in their real-time interactions. The optimization of dynamic response strategies plays central role in multiagent decision systems and problems of this type are manifested in a wide range of applications, such as reconnaissance of multiple uninhabited aerial vehicles (UAVs), search mission of various rescue teams in a natural disaster (Cao et al. 2024), patrol schedule of multiple troops in a security protection (Xie et al. 2020), and collaborative pursuit-evasion game (Wang et al. 2020) and so on.

To solve the challenging problem on optimizing response strategies, several agents’ decision models have been developed in the past decades, including decentralized partially observable Markov decision process (POMDP) (Seuken and Zilberstein 2008), interactive POMDP (Gmytrasiewicz 2005), interactive dynamic influence diagrams (I-DIDs) (Doshi et al. 2009) and so on. Among these models, I-DIDs demonstrate a generalization technique of modeling either collaborative or competitive agents, and computational advantages in solving the problems (Zeng and Doshi 2012; Doshi et al. 2020). More importantly, I-DIDs inherit structural representations of probabilistic graphical models, making them inherently explainable for reasoning about other agents’ behaviors. This enables us to explicitly examine the influence of other agents’ unknown behaviors on the subject agent’s response strategies. Hence, this article investigates I-DIDs for optimizing the subject agent’s decisions in the response to unknown behaviours of other agents.

Most of the existing I-DID research has focused on improving the planning scalability through a number of approximate algorithms that are inspired by the concept of minimal mental models and type equivalence (Pynadath and Marsella 2007; Albrecht et al. 2020; Doshi et al. 2020; Pan et al. 2022). However, this line of I-DID research still operates under the assumption that the subject agent must postulate true behaviors of other agents within a limited set (Pan et al. 2021). One natural idea is to generate a sufficiently large set of candidate behaviours that could ideally contain unexpected behaviours of other agents - theoretically, the behavioural space of other agents is uncountable. This becomes computationally impossible for the conventional I-DID solutions since the existing methods can only focus on a limited set of models with determined beliefs - failing to capture unexpected behaviours (Pan et al. 2023). Although the limited behavioural space provides computationally feasible solutions, it leads to serious consequence when a subject agent faces unknown behaviours of other agents.

Inspired by evolutionary mechanisms, Zeng Zeng et al. (2022) have recently shown promising results on exploiting a genetic algorithm (GA) to model unexpected behaviours of other agents in I-DIDs. This research is followed by the GA-based solution to the I-DIDs (Pan et al. 2023). However, the GA-based solutions compose the set of candidate behaviours by generating individual behaviours in a separate way. The techniques may ignore relations among potential behaviours that would be actually executed by the same agent in a same environment. The behavioural difference only lies in the agent’s initial beliefs (views) on the environment. Hence we need to balance individual and collective behaviours in a new solution. To develop a set of candidate behaviours that simulate the agents’ behaviours in a more precise way, we are motivated to exploit swarm intelligence (SI) (Smith 2020) based solutions to I-DIDs. This is to exploit the potential of the SI algorithms on generating collective, diverse behaviors for other agents that are to be modeled by a subject agent in the I-DIDs.

In this article, we align with the trajectory of swarm intelligence research and incorporate two distinct techniques to improve I-DID solutions: a particle swarm optimization algorithm (PSO (Kennedy and Eberhart 1995)) and an ant colony optimization algorithm (ACO (Dorigo et al. 2006)). Generally, both PSO and ACO algorithms exhibit strong potential in escaping local optima and exploring a wide range of behaviors over iterations. Considering agents’ behavioral representation, we design novel rules to compute particles’ position and velocity in PSO, and propose a new method for updating ants’ positions in ACO. These implementations ensure the rationality of new behaviors, assessed through decision-making principles, while enhancing behavioral diversity. Our main contributions are summarized below.

- We adapt two SI algorithms to solve I-DIDs, namely the PSO/ACO-enabled behaviours (PSB/ACB to be formulated formally in Alg. 1-2). This new research will elicit a new line of I-DID research through evolutionary computation and inspire new ideas of solving a dynamic response strategy optimization problems.
- We analyze the I-DID solution quality due to randomness of the evolutionary operations in a theoretical way. This provides the clue of adjusting the algorithms and confidence about the algorithm performance.
- We empirically compare the PSB/ACB algorithms with the state-of-art I-DID solutions (Zeng et al. 2022; Pan et al. 2023) in two problem domains and explore improvement of the new algorithms.

We do not aim to invent a new type of SI techniques, but to adapt two typical SI methods to solve the dynamic response strategy optimization problem in I-DIDs. This is also the first time of developing SI-based I-DID techniques and solving the challenging problem of unexpected behaviours in I-DIDs. The new I-DID approaches provide a good coverage of potential behaviours of other agents according to which a subject agent is equipped with sufficient capability to deal with unexpected interactions in a real-time manner. It removes the barrier of knowledge-based approaches for hypothesizing the potential behaviours when the I-DID model shall be constructed in a decision optimization problem. Hence the I-DID models become more accessible in a general application where little domain knowledge is known and behaviours of other agents becomes more unpredictable.

This article is organized as follows. Section 2 provides background knowledge on I-DIDs. Subsequently, Sect. 3 presents two SI-based approaches to generate new behaviours of other agents in I-DIDs with theoretical analysis. Section 4 shows the experimental results of the new algorithms in the comparison. Finally, we conclude this research and discuss future work in Sect. 5.

## Background knowledge and related works

We first provide the background knowledge of I-DIDs solutions to a dynamic response strategy optimization problem, and then review the latest research.

### Interactive dynamic influence diagrams (I-DIDs)

**Fig. 1:**
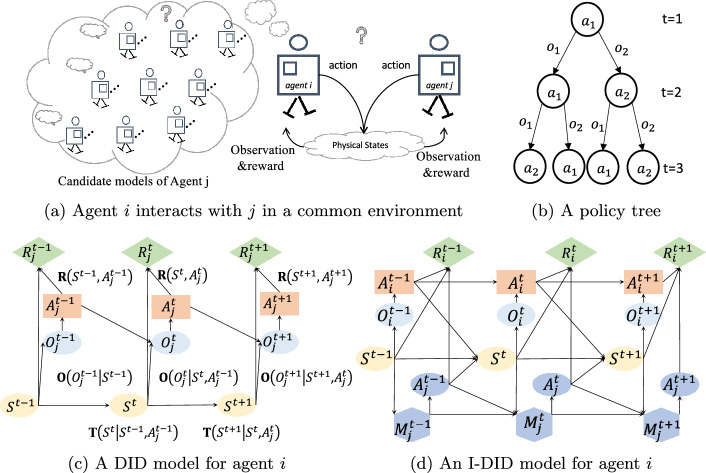
An MSDM scenario involving two agents: agent *i* models agent *j* in an I-DID model of three time steps (*T*=3)

I-DID is a probabilistic graphical model of solving agents’ decision problems from the viewpoint of individual agents. It explicitly represents how one subject agent optimizes its response strategy during its interactions with other agents in a common environment. This is different from other approaches that solve the multiagent decision problem from the external perspective of multiple agents. For example, adaptive dynamic programming and reinforcement learning offer good scalability while lacking an explicit representation of how the decision is optimized. Game theory based approaches offer a Nash equilibrium based solution, which is subject to common knowledge assumption (Doshi et al. 2009). The challenge of solving the response strategy optimization problem lies in the uncertainty of other agents’ behaviours that are modelled in I-DID. We elaborate the decision making scenario of two agents (the subject agent *i* and the other agent *j*) and agent *i*’s I-DID model in Fig. 1.

In Fig. 1(*a*), the subject agent *i* does not know the true environmental states (*S*) and can not directly observe what the other agent *j* acts ($A_j$) during their interaction, which impacts *i*’s decisions ($A_i$) and reward values (*R*), and shall act given what it believes about physical environments and *j*’s behaviours - a series of actions upon observations (*O*) over a number of time steps. Agent *i* updates its belief according to its observations at each time step *t*. To estimate the agent *j*’s behaviours, agent *i* hypothesises a set of candidate models for agent *j* that could be either a decision model or a behavioural model. An I-DID model uses a dynamic influence diagram (DID) (Tatman and Shachter 1990) as the decision model for the other agent *j* (in Fig. 1 (*c*)). The solution of the dynamic influence diagram is a policy tree (in Fig. 1 (*b*)) that describes *j*’s optimal actions upon every observation at each time step, which is generally named as the agent *j*’s behavioural model. For example, Fig. 1 (*c*) is a DID with three time steps where the probability distributions $Pr(S^t|S^{t-1}, A^{t-1})$, $Pr(O^t|S^{t}, A^{t-1})$ and $R(S^t, A^t)$ are the transition, observation and reward functions as formulated in POMDP (Kaelbling et al. 1998). For example, $Pr(S^t|S^{t-1}, A^{t-1})$ is the probability of being in the state $S^{t}$ when the action $A^{t-1}$ is executed on the previous state $S^{t-1}$. Once a DID model is built, we can use conventional inference algorithm to solve the model and obtain the optimal policy (behavioural model) for the agent (Zeng and Doshi 2012).

An I-DID model extends a single-agent DID into a multiagent decision model. It introduces a new node (the $M_j$ node in Fig. 1(*d*)) that contains a set of agent *j*’s candidate models that are DIDs with different parameter values. The set of DIDs provide various types of *j*’s behaviours that potentially contain its true behaviours. In addition, I-DIDs have a new model update (the dotted line) that encodes how agent *i* updates its belief over *j* models over time. Since agent *i* does not observe *j*’s actions directly, agent *i* needs to update its belief once it receives new observations after each time step. This leads to the exponentially growing number of candidate models of *j*’s models (in $M_j$) over times since every candidate model needs to be updated based on *j*’s observations.

Once we solve agent *j*’s candidate decision models, we represent their policies in the model node. Subsequently, the I-DID becomes a conventional DID and can be solved through the DID algorithms for generating agent *i*’s optimal policies. However, the I-DID solutions assume that the true models of agent *j* is within agent *i*’s candidate model space so that agent *i* can correctly update its beliefs about physical states and *j*’s models over times. The agent *i*’s optimal policies will fail when any unexpected behaviour of agent *j* occurs in their interactions. In this article, we aim to generate a limited set of agent *j*’s candidate models that hold a good chance of containing agent *j*’s true models.

Meanwhile, we notice that the behavioural model can also be built directly without building a decision model, e.g. learning agents’ policies from historical data recording their interactions (Conroy et al. 2015; Pan et al. 2021). The data-driven I-DID solutions become attractive particularly when agent *i* does not need to learn how agent *j* optimizes its decisions. In this article, we will use behavioural models to represent agents’ policies and aim to generate new behaviours through SI-based approaches.

### Related Works

Evolutionary based approaches have shown promising results of optimizing response strategies (Byrski et al. 2015). For example, Eker and Akın (2013) developed genetic algorithms for solving dec-centralized POMDP while Klijn and Eiben (2021) proposed deep coevolutionary algorithms to improve multiagent reinforcement learning techniques. Zeng et al. (2017) adapted memetic algorithms to improve human-like behaviours of agents’ interactions while Hou et al. (2023) differentiated agents’ role in the improved multiagent reinforcement learning. Rosaci and Sarna (Rosaci and SarnÃ 2013) develops a proactive cloning process to improve agents’ performance for users’ interest. Meanwhile, agents-based evolutionary algorithms have been well developed to solve complex optimization problems (Li et al. 2023). Most of this research exploits a distributed agent decision making framework to improve the capability of evolutionary approaches. Coping with unknown behaviours is a grand challenge to agent research while most of the work resort to traditional AI solutions, e.g. theory of mind, decision theory and so on (Marcus 2020). For example, Pynadath and Marsella (2007) used the concept of mental model to reduce uncertainty of candidate models ascribed to other agents. Rabiee et al. (2021) used meta-reasoning techniques to aid autonomous planning and mitigate negative impacts of plan failure due to incomplete opponent knowledge. Papoudakis and Albrecht (2020) developed variational auto-encoders for opponent modeling, reducing the information needed for agent planning.

Similarly, most of the I-DID solutions used the concepts of behavioural and value equivalences to reduce the candidate model space of other agents (Pan et al. 2021). In principle, policy trees of other agents are to be merged if they have identical impact on a subject agent’s decision. This line of I-DID research still assumes that true behaviours of other agents are contained in the subject agent’s model space. Essentially, the subject agent can’t update its beliefs over other agents’ models since the unexpected behaviours are not encoded in the I-DID model. It has been examined in general opponent modeling (Stephen 2020).

Recently, Wang et al. (2021) used evolutionary game theory to improve agents’ response strategies where users’ behaviours are unpredictable in social media. (Pan et al. 2023) proposed a GA-based framework to generate new behaviours of other agents by injecting randomness into opponent modeling. The evolutionary approaches show the potential to modeling unknown behaviours in I-DIDs, and opens up a new direction of modeling other agents in I-DIDs. In this article, we investigate the evolutionary I-DID solutions in a systematic way (also empirically comparing two relevant SI based I-DID solutions) and expect to elicit a new wave of evolutionary agent planning research.

## New behaviour generation algorithms

Essentially, we need a new way to generate a good set of candidate behaviours of other agents. The interacting behavioural system resembles interactive agents that act and response simultaneously in the SI interaction. In this section, we will apply two SI approaches to generate a new set of behaviours for other agents. Given an initial set of candidate behaviours, the behaviours evolve through the SI update operations and may produce true behaviours of other agents. By doing this, we can build an I-DID model for the subject agent *i* who represents behaviours of the other agent *j* through the SI-based approaches.

### Formulation of the behavioural optimization problem

As elaborated in Fig. 1(*d*), a behaviour $\sigma$ can be represented by a policy tree of *T*-time steps $\mathcal {H}^T = \bigcup h^T$ where $h^T$ is a sequence of action-and-observation $h^T = \{a_1, o_1, \cdots , o_{T}, a_T\}$.

1 $$\begin{aligned} \begin{array}{l} F(\mathcal {H}^T) = \sum _{\forall s \in S} b^0(s) V_{\mathcal {H}^T}(s) \end{array} \end{aligned}$$

where $b^0(s)$ denotes the agent’s initial belief over state *s* at time $t=0$, and $V_{\mathcal {H}^T}(s)=R(s,a(\mathcal {H}^T))+\lambda \sum _{\forall s' \in S} Pr_{a(\mathcal {H}^T)}(s'|s)[\sum _{\forall o \in O} Pr_{a(\mathcal {H}^T)}(o|s') V_{o(\mathcal {H}^T)}(s')]$ denotes the expected reward for state *s* under a specific behaviour $\mathcal {H}^T$ for any state $s \in S$, where *Pr*() and *Pr*(|) represent the probability function and conditional probability function respectively, $a(\mathcal {H}^T)$ is the corresponding action sequence of the behaviour $\mathcal {H}^T$ and $o(\mathcal {H}^T)$ represents observation sequence of the behaviour $\mathcal {H}^T$ respectively.

To compute the expected reward $F(\sigma )$, we input the behaviour $\sigma$ (the policy tree $\mathcal {H}^T$) as the evidence in the action and observation nodes of the corresponding DIDs. For example, the actions *a*(*t*) and observations *o*(*t*) instantiate the decision nodes *A* and chance nodes *O* respectively in Fig. 1 (*c*). The computation can be done efficiently by a well-developed decision tool, e.g. GeNIe (Druzdzel 1999) and HUGIN (Andersen et al. 1989).

To increase the coverage of new behaviours over potentially unexpected behaviours, we aim to generate a set of *K* behaviours each of which is the optimal solution to an agent *j*’s DID model associated with a specific belief (a probability distribution over the initial physical state *S*). The *K* value is the largest number of candidate models of other agents.

Given a dataset of possible behaviours $\mathcal {D}^T = \bigcup \mathcal {H}^T$, we aim to select *K* behaviours that yield the maximum expected reward. Our objective is to maximize the diversity across the chosen *K* behaviours, formulated as:

2 $$\begin{aligned} \begin{array}{l} max_{\mathcal {D}_K^T}~~ diversity(\mathcal {D}_K^T)\\ s.t.~~ \mathcal {D}_K^T \subset \mathcal {D}^T \end{array} \end{aligned}$$

3 $$\begin{aligned} diversity(\mathcal {D}_K^T)&= |\mathcal {D}_K^T| \times \frac{|\bigcup _{\mathcal {H}^T\in \mathcal {D}_K^T} {\bigcup _{ t_s \in \mathcal {H}^T} t_s|}}{\sum _{\mathcal {H}^T\in \mathcal {D}_K^T}{|\bigcup _{t_s \in \mathcal {H}^T}t_s|}} \end{aligned}$$

where $\mathcal {H}^T\in \mathcal {D}_K^T$ is the policy tree, $t_s$ is the sub-tree of $\mathcal {H}^T$, $\bigcup _{t_s \in \mathcal {H}^T}$ finds the unique sub-trees in $\mathcal {H}^T$ and $|\mathcal {D}_K^T|$ denotes the size of policy tree set $\mathcal {D}_K^T$.

**Fig. 2:**
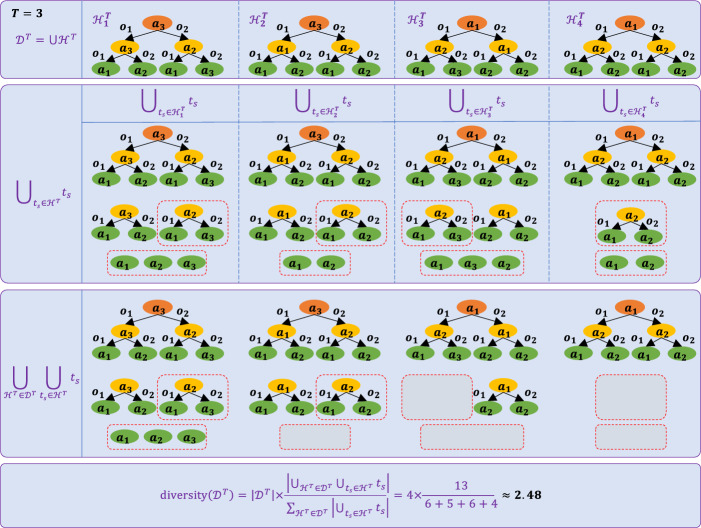
The process of computing behavioral diversity. Subtrees that appear repeatedly are marked with a red dashed border and gray squares. These redundant subtrees are eliminated during the union operation on the subsets

As illustrated in Fig. 2, the process of computing behavioral diversity involves identifying and eliminating redundant subtrees. Subtrees that appear multiple times are highlighted with red dashed borders, indicating their repetition. These redundant subtrees are removed to ensure that only unique subtrees are retained in the union set $\bigcup _{\mathcal {H}^T \in \mathcal {D}_K^T} \bigcup _{t_s \in \mathcal {H}^T} t_s$, which is also highlighted with red dashed borders and gray squares.

The behavioural space - the number of the policy trees $(|A||\Omega |)^T$ where |*A*| and $|\Omega |$ are the action and observation sizes respectively - increases exponentially with the planning horizon *T*. More seriously, we are to find optimal behaviours for all possible beliefs. In theory, there are uncountable beliefs in the search space. Consequently, the potential solution space is extremely complex in the new behavioural optimization.

As shown in the existing I-DID techniques, solving a single DID with a specific belief is computationally expensive. The new optimization problem of *K* behaviours is much more complicated and cannot be solved by the state-of-art I-DID techniques. Hence we aim to adapt two typical SI-based approaches below and solve the new I-DID problem.

### Generation of new behaviours through PSO

Particle swarm optimization (PSO) is an algorithm inspired by the birds’ predation, and illustrates that a complex behaviour can be formed by the interaction of a set of simple rules (Kennedy and Eberhart 1995). The performance depends on a swarm search strategy and information exchange among individuals. Given the PSO mechanism, we aim to generate a new set of agent’s behaviours upon its initial behaviours. We extract a sequence of actions from a policy tree $\mathcal {H}^T$ and consider it as the particle position $p=\bigcup a_l$, where $a_l \in A$ and *l* is the position index of the action $a_l$. The size of the particle position |*p*| is up to $\frac{|\Omega |^T - 1}{|\Omega | -1}$. For example, the particle position is $p=(a_1, a_1, a_2, a_1, a_2, a_1, a_2)$, where *l* has the maximum value 7, since there are 7 actions from the policy tree.

We then define the particle velocity as a set of three-tuple $v=\bigcup (l,a_l,Pr(a_l))$, where *l* is the action’s index in the particle location, $a_l$ is the action (corresponding to the action in the particle position) and represents the value of the particle location, and $Pr(a_l)$ is the probability of the action $a_l$ changed by the particle. Subsequently, we use a two-tuple $\sigma$=(*p*, *v*) to represent a particle with two main components: the position *p* and the velocity *v*. Updating the particle involves the calculation of *p* and *v*. Since behavioral representations cannot be calculated as real numbers, unlike in conventional PSO operations, we define four arithmetic rules for updating particle position and velocity in two typical scenarios.

**[Situation 1]** Given the current position $p=\bigcup a_l$, the particle performs a random walk to the target position $p'=\bigcup a'_l$ with the velocity $v=\bigcup (l,a_l,Pr(a_l))$. The velocity and position are calculated below:

- The *minus* operator $\ominus$ between the particles’ positions to calculate the particle velocity: $v\leftarrow p'\ominus p$ where $a_l\leftarrow a'_l$, $$Pr(a_l)\leftarrow {\left\{ \begin{array}{ll} 1/|A|,\quad & a'_l \ne a_l \\ 0,\quad & a'_l = a_l \end{array}\right. }$$. If $Pr(a_l)$ is equal to zero, we can remove the element $(l,a_l,Pr)$ from *v*.
- The *plus* operator $\oplus$ to calculate a new particle position: $p \leftarrow p' \oplus v$ where $$a_l\leftarrow {\left\{ \begin{array}{ll} a'_l,\quad & r\le Pr(a_l) \\ a_l,\quad & r> Pr(a_l) \end{array}\right. }$$, *r* is a random number.

**[Situation 2]** Given the scalar $\omega$, the particle walks towards the new position with $v=\bigcup (l,a_l,Pr)$ and the weight $\omega$ - leading to the weighted velocity $v'$.

- the *times* operator $\otimes$ for the velocity *v* and the scalar $\omega$: $v' \leftarrow \omega \otimes v$ where $v'=\bigcup (l,a_l,min(\omega Pr, 1))$.
- the *merging* operator $\uplus$ for the two velocities - *v* and $v^*$: $v' \leftarrow v \uplus v^*$ If $a^*_l$ is equal to $a_l$, we add the element $(l,a^*_l, min(Pr_l+Pr^*_l, 1))$ into $v'$; otherwise, we add the elements $(l,a^*_l, Pr^*_l)$ and $(l,a_l, Pr_l)$ into $v'$.

where the operator $\otimes$ has a higher priority than other operations and parentheses $(\cdot )$ with the highest priority. We also present the implementation of the four arithmetic rules in the Supplementary Materials (please refer to Algorithm 1).

As one particle is one potential policy for agent, we use the expected reward of the behaviour ($F(\sigma )$ in Eq. 1) as its fitness value. We compute the fitness value $F(\sigma )$ through the GeNIe tool.

Similar to a general evolutionary algorithm, PSO needs to update a particle during each iteration. The update uses the four operators to calculate the new position and velocity of the particle as follows.

4 $$\begin{aligned} v_{n+1}&=\omega \otimes v_n \uplus c_1 \otimes (\bar{p}_n \ominus p_n) \uplus c_2 \otimes (p^*_n \ominus p_n)\end{aligned}$$

5 $$\begin{aligned} p_{n+1}&=p_n \oplus v_{n+1} \end{aligned}$$

where $v_n$ and $p_n$ are the particle velocity and position respectively at the *n*-th iteration. $\bar{p}_n$ is the position of the local best particle $\bar{\sigma }$ with the best fitness at the *n*-th iteration, $p^*_n$ is the position of the global optimal particle $\sigma ^*$ with the best fitness up to the *n*-th iteration, and $\omega$ is the inertia weight, which indicates how much the particle inherits from the original velocity. The learning rates $c_1$ and $c_2$ decide how fast the particle will learn from the best behaviours of the local optimal particle $\bar{p}$ and the global optimal particle $p^*$ respectively, and $\omega$ controls the speed at which the particle remains in place. The particle swarm optimization algorithm searches for the global optimum through iterative updates of the best positions. This process requires continuous exploration of the search space. Even with the known global optimal particle, it does not guarantee that the algorithm will converge to the optimal solution. Therefore, it is necessary to assign appropriate weights to the current optimal particle in each iteration, gradually converging towards the best solution.

**Algorithm 1:**
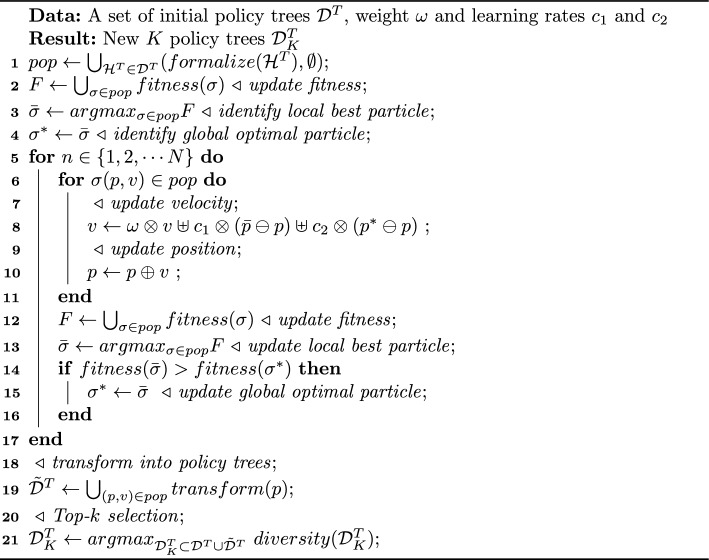
PSO-Enabled behaviours (@*PSB*)

Accordingly, we describe the general PSO-enabled behaviour (PSB) generation process in Alg. 1 and present a general PSO-enabled behaviour (PSB) generation framework in the Supplementary Materials (please refer to Figure 1). We initialize a population of particles *pop* by converting every known policy tree into a particle $\sigma =(p,v)$ (line 1). By computing the fitness value of every particle $F(\sigma )$ (line 2), we identify the local and global best particles $\bar{\sigma }$ and $\sigma ^*$ respectively (line 3-4). Then, during *N* iterations, we update the position and velocity of every particle in the population (line 6-11). Meanwhile, we update the particle’s fitness using the *fitness* operator (line 12). We update the local and global best particles $\bar{\sigma }$ and $\sigma ^*$ respectively  (line 11-16). In each iteration, the particle moves towards the position of either a global optimal particle $\sigma ^*$ or a local optimal particle $\bar{\sigma }$ (line 5-17). Finally, the particles are converted into a set of policy trees and decoded into new behaviours via *transform* operator  (line 18-19). The *K* new behaviours are retrieved from the top-*K* ones in terms of their fitness values in the final population (line 20-21). The individual behaviours have different initial beliefs and are optimized over the iteration.

### ACO adaptation for new behaviours

An ant colony algorithm (ACO) is a bionic optimization technique that simulates the foraging behaviour of ant colonies in nature (Dorigo et al. 2006). Similar to the PSB algorithm, we adapt the ACO algorithm to generate new behaviours for an agent. An ant maintains a series of positions each of which is one action from a policy tree. Let denote the ant as $\zeta =(a_1, \cdots , a_l)$, where $a_l$ is an agent’s action $a_l \in A$, $l \in (1, \cdots , L)$, and $L=\frac{|\Omega |^T - 1}{|\Omega | -1}$ is the total number of ordered actions (from *t*=1 to *T*) in a policy tree. For example, the ant takes the actions $(a_1, a_1, a_2, a_1, a_2, a_1, a_2)$ following the policy tree in the left and the action order is immutable.

**Algorithm 2:**
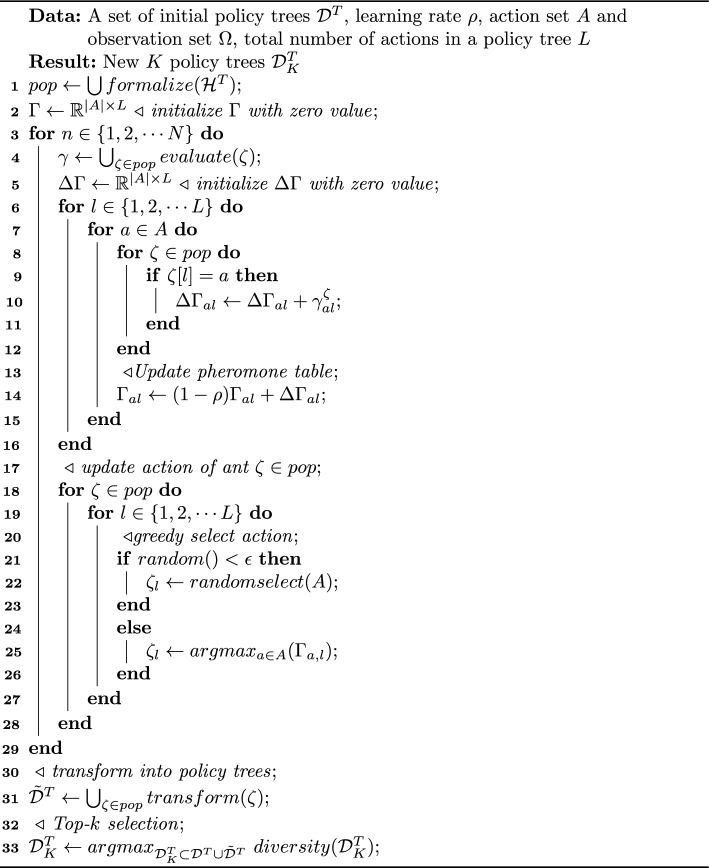
ACO-Enabled Behaviours (@*ACB*)

Naturally, an ant is the holder of the policy as encoded in the ordered actions over the positions. When the ant $\zeta$ performs the action *a* ($\in A$), it generates the pheromone $\gamma ^\zeta (a,l)$ for the action *a* at the position *l*. In Eq. 6, we compute the pheromone value of the action *a* at the position *l* as the expected reward, namely *ER*(*a*, *l*), received by an agent when the corresponding policy is followed.

6 $$\begin{aligned} ER(a,l)&= \sum _{s \in S} b^t(s)R(s,a) \end{aligned}$$

where $b^t(s) = \frac{Pr(o|s,a\sum _{s'\in S}{Pr(s|s',a)}Pr(s'|b^{t-1})}{\sum _{s's''\in S}{Pr(o|s'',a)Pr(s''|s',a)Pr(s'|b^{t-1})}}$ is the agent’s belief at the time step *t*, which is updated from the agent’s initial belief $b^0(s)$.

We maintain pheromone values $\Gamma _{a,l} = ER(a,l)$ for each action in every position, updating these values when ants perform new actions. The pheromone values are represented in a $|A| \times L$ table and iteratively updated using Eq. 8.

7 $$\begin{aligned} \Delta \Gamma ^{n+1}_{a,l}&= \sum _{\zeta \in pop} \gamma ^{\zeta }_{a,l} \end{aligned}$$

8 $$\begin{aligned} \Gamma ^{n+1}_{a,l}&= (1-\rho )\Gamma ^{n}_{a,l} + \Delta \Gamma ^{n+1}_{a,l} \end{aligned}$$

where $\gamma ^{\zeta }_{a,l}$ is the pheromone left by the ant $\zeta$ at the position *l* with the action *a*, $\Delta \Gamma ^{n+1}_{a,l}$ is the pheromones collected by the ant when it performs the action *a* at the position *l*, and $\Delta \Gamma ^{n}_{a,l}$ is the increased pheromone for the action *a* at the position *l* in the *n*-th iteration. Meanwhile, $\rho$ represents the volatile factor and $1-\rho$ is the residual factor in the pheromone update.

The parameter $\rho$ partially decides the search space therefore impacting the solution convergence. If $\rho$ is too small, a large amount of pheromone could still remain on each path, which will lead to many invalid paths therefore reducing the algorithmic convergence speed. On the contrary, if $\rho$ is too large, a valid path would not be abandoned.

We introduce a general ACO-based behavior (ACB) generation framework in Alg. 2 and present details in the Supplementary Materials (see Figure 2). Initializing ants and their pheromone tables (lines 1-2), we update these by assessing each ant’s actions per iteration (lines 7-16). Ants select next actions using an epsilon-greedy approach guided by updated pheromones (lines 17-30). The process ends after *N* iterations (lines 3-29). Finally, we convert the ant population into policy trees and select the top *K* behaviors (lines 30-33). Both PSB and ACB algorithms share four core operators detailed in the Supplementary Materials (see Algorithm 2).

### Algorithmic analysis

We run both the PSB and ACB algorithms over *N* iterations, and show the convergence of the population in terms of individuals’ diversity (to be defined in the experiments). Inheriting from the solution quality of the PSO and ACO algorithms, the new algorithms could generate the global or local optimal behaviours in terms of their expected rewards. However, this is not guaranteed due to the PSB/ACB operational randomness. Since we will use new behaviours for agent *j* in the agent *i*’s I-DID model, we take a further step to analyze their impact on the agent *i*’s expected rewards.

Let the true and new behaviours of the other agent *j* be $\mathcal {H}$ and $\mathcal {H}^*$ respectively. Accordingly, we have the difference between the predicted actions at each time step *t* from these behaviours $\Delta _j=|Pr(a_j^t|\mathcal {H})-Pr(a_j^t|\mathcal {H}^*)|$. Both the PSB and ACB algorithms expect to reduce the difference over *N* iterations. However, due to the randomness of the evolutionary operators, e.g. the *r* and $\epsilon$ parameters in the PSB and ACB algorithms respectively, the algorithms will spend much more iterations $\frac{N}{r}$ (or $\frac{N}{\epsilon }$) to develop the convergence.

Since the algorithms involve the randomised operators, we expect to bound the convergence of the new behaviours towards the true ones with some confidence. We refer the probable convergence to the *Hoeffding* inequality on the proof of the bounded sample complexity in a probabilistic estimation. Accordingly we can bound the sum of the difference between the probabilities of the new and true behaviours in a probable convergence rate in the proposition below.

#### Proposition 1

9 $$\begin{aligned} Pr(T\sigma \le \varphi ) \ge 1- |A_j|T{e^{-2\frac{TN{\varphi }^2}{r|A_j|^2}}} \end{aligned}$$

where $|A_j|$ is the size of agent *j*’s actions, *T* is the planning horizon and $\sigma$ is the worst case of predicting *j*’s actions and equal to $\underset{t}{max}\sum _{a_j} |Pr(a_j^t|{\mathcal {H}}_{j}) - Pr(a_j^t|{\mathcal {H}}_{j}^*)|$, and $\varphi$ is the upper bound. *r* is the PSB parameter (equivalent to $\epsilon$ in ACB).

#### Proof

We let $\Delta _j=|Pr(a_j^t|\mathcal {H})-Pr(a_j^t|\mathcal {H}^*)|$ be the difference between the predicted actions by the PSB/ACB algorithms and the actions from the true behaviours at each time step *t*. $Pr(a_j^t|\mathcal {H})$ is the sample mean since the behaviours are generated by different evolutionary operators over iterations while $Pr(a_j^t|\mathcal {H}^*)$ is the true mean. We use the *Hoeffding’s inequality* to bound the convergence of the sample mean to the true mean through the probable rate.

$$\begin{aligned}&Pr(|Pr(a_j^t|\mathcal {H}) - Pr(a_j^t|\mathcal {H}^*)| > \hat{\epsilon }) \le {e^{-\frac{N}{r}T\hat{\epsilon }^2}} \end{aligned}$$

However, we need to calculate every action in each step and the total number of steps is *T*

$$\begin{aligned}&Pr(\sum \limits _{t}\sum \limits _{a_j}|Pr(a_j^t|\mathcal {H}) - Pr(a_j^t|\mathcal {H}^*)| > |A_j|T\hat{\epsilon }) \le |A_j|T{e^{-2\frac{N}{r}T\hat{\epsilon }^2}} \end{aligned}$$

To simplify the notation, we let $\varphi = |A_j|\hat{\epsilon }$, and get the below.

$$\begin{aligned}&Pr(\sum \limits _{t}\sum \limits _{a_j}|Pr(a_j^t|\mathcal {H}) - Pr(a_j^t|\mathcal {H}^*)| > \varphi ) \le |A_j|T{e^{-2\frac{TN{\varphi }^2}{r|A_j|^2}}} \end{aligned}$$

$$\begin{aligned}&Pr(\sum \limits _{t}\sum \limits _{a_j}|Pr(a_j^t|\mathcal {H}) - Pr(a_j^t|\mathcal {H}^*)| \le \varphi ) \ge 1- |A_j|T{e^{-2\frac{TN{\varphi }^2}{r|A_j|^2}}} \end{aligned}$$

Let $\sigma = \underset{t}{max}\sum _{a_j} |Pr(a_j^t|{\mathcal {H}}_{j}) - Pr(a_j^t|\mathcal {H}^*)$ be the most case of predicting the actions through the algorithms, we have

$$\begin{aligned}&\sigma \ge \sum \limits _{a_j}\Delta _j \end{aligned}$$

Hence, $T\sigma$ has the probability at least $1- |A_j|T{e^{-2\frac{TN{\varphi }^2}{r|A_j|^2}}}$ to approach the upper bound $\varphi$. **[END]**

We make a further step to investigate how the agent *j*’s behavioural difference would impact the agent *i*’s expected rewards, which is the ultimate goal of exploring new behaviours of other agents. Let $\Upsilon _i = |ER(m_{i}) -ER(m^*_{i})|$ where $ER(m_{i})$ is the agent *i*’s expected rewards when its I-DID model is built using agent *j*’s new behaviours from either the PSB or ACB algorithm, and $ER(m^*_{i})$ is the agent *i*’s expected rewards when the true behaviours of agent *i* are used in the agent *i*’s I-DID model. Through the process (Chen et al. 2015), we can bound the error of the agent *i*’s expected rewards through the worst case of using the new behaviours of agent *j* as follows.

10 $$\begin{aligned} \Upsilon _i \le \sigma R_i^{max}[(T-1)(1 +3(T-1)|\Omega _i||\Omega _j|) + 1] \end{aligned}$$

where $R_i^{max}$ is the agent *i*’s largest reward in the I-DID model and $|\Omega _i|$ ($|\Omega _j|$) is the size of agent *i*’s observations.

Due to the algorithm randomness, new behaviours of agent *j* will be deviated from its true behaviours and the difference has seriously impacted agent *j*’s expected rewards. We will demonstrate such influence in the experiments. $\square$

## Experimental results

The PSB/ACB generated behaviours will serve as potential behavioural models of agent *j* to be embedded in agent *i*’s I-DID models. The I-DID model is solved to obtain agent *i*’s policy and agent *i* uses the policies to interact with agent *j* in the experiments. The purpose of the experiments is to demonstrate how the PSB/ACB-generated behaviours of agent *j* could improve the agent *i*’s I-DID models so as to optimize agent *i*’s decisions under uncertainty. We evaluate the policies in terms of the average rewards that agent *i* receives when it interacts with agent *j* over a number of times. We compare the following four algorithms to solve I-DIDs.

- The I-DID algorithm (Exact (Pan et al. 2021)) that expands agent *j*’s candidate models following the model update link in Fig. 1. We use Exact as a baseline which has been regularly in all the existing I-DID algorithm comparisons.
- The genetic algorithm (GA) (Zeng et al. 2022; Pan et al. 2023) develops candidate models for agent *j* utilizing genetic manipulations. It represents policy trees as chromosomes, iteratively evolving behaviors via selection, mutation, and crossover, reflecting the pinnacle of I-DID solutions adapted for unknown agent behaviours. See Ev-IDID for details $^1$.
- The PSO-enabled algorithm to provide agent *j*’s candidate models (PSB) in Alg. 1.
- The ACO-enabled algorithm to provide agent *j*’s candidate models (ACB) in Alg. 2.

To evaluate the algorithm performance over iterations, we compute the average fitness value (Avg. Fitness) of all the individuals in the PSB/ACB algorithms. In addition, we develop one diversity measurement (in Eq. 3) to evaluate the difference of policies generated by the PSB/ACB algorithms. Following the existing I-DID research (Albrecht et al. 2020; Pan et al. 2023), we evaluate all the algorithms on two classical multiagent planning problems: a multiagent tiger problem and a multiagent unmanned aerial vehicle (UAV) problem. Experiments are conducted on Windows 10 with an Intel(R) Core(TM) i7-6700 @3.40GHz CPU and 24 GB RAM. Due to the space constraint, we concisely present comparative studies accompanied by statistical analysis, highlighting the frameworks of PSB and ACB, their distinct operators, and common functionalities in the supplementary material. For extensive access to all the implementations, datasets, and vital resources related to the SI-IDID framework, please refer to our GitHub repository at SI-IDID ,^1^ the new extension of the Ev-IDID $^1$ toolkit.

### The multiagent tiger problem domain

**Fig. 3:**
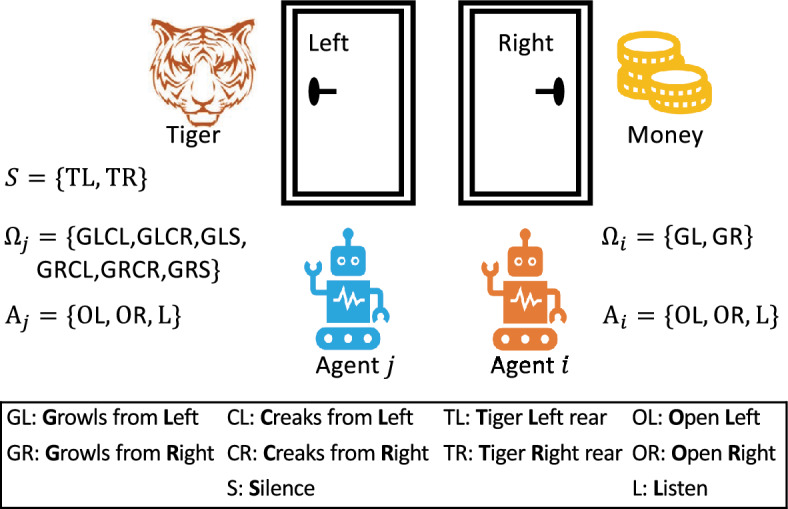
The two-agent tiger problem domain: the subject agent *i* optimizes its behaviours by predicting agent *j*’s behaviours when it decides the *open the door* option

The multiagent tiger problem is a classic multiagent planning problem and we consider the version of two-agents first in this section and explore more complicated ones in the scalability discussion. As shown in Fig. 3, two agents, agent *i* and *j*, are facing two doors behind of which either a pot of gold exists or a fierce tiger is waiting. They have three actions: open the left door (OL), open the right door (OR), or listen (L) upon their observations. The agent can’t see the actions of the other, but hear the sounds that are due to either the tiger’s growls or the creak of a door opened by the other. Both of them get rewarded by the gold; otherwise, he/she is to be eaten by the tiger if only one of them opens the door where the tiger hides.

There are two states ($|S|=2$) and three available actions ($|A_i|=|A_j|= 3$) for both agents. As we build the I-DID models from the viewpoint of the subject agent *i*, it receives six observations ($|\Omega _i| =6$) while the number of the agent *j*’s observations is three ($|\Omega _j| = 3$). We assume that agent *j* does not model agent *i*. The candidate models of agent *j* are provided by either exactly expanding its initial models (Exact) or through the evolutionary algorithms, e.g. GA, PSB or ACB. We built the I-DID models for agent *i* and represented agent *j*’s potential behaviours in the models. Solving the I-DIDs returns agent *i*’s policies that are used to be executed when agent *i* interacts with the other agent *j*. The agent *j*’s policies are either drawn from the potential behaviours or randomly generated from the existing ones.

**Fig. 4:**
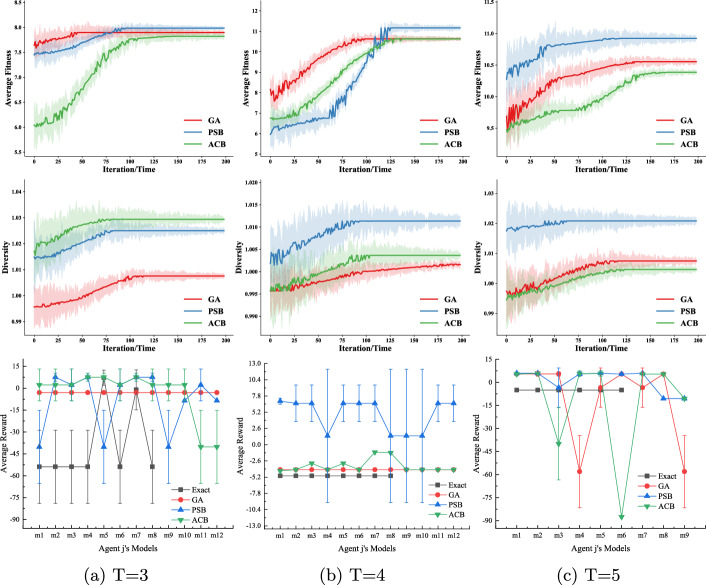
This evaluation compares four methods over 20 runs, focusing on convergence, diversity, and average rewards when the candidate set includes the true model within I-DIDs. Experiments started with 8 initial models, maintained a population of 8, and allowed up to 200 iterations. After convergence, top-12 models were selected for analysis. We considered *T*=3, 4, 5. Optimal settings for PSB and ACB are in Table 1

#### Average fitness values/diversity v.s. average rewards

The upper panel of Fig. 4 shows the convergence of the average fitness values when the GA, PSB and ACB algorithms generate agent *j*’s behaviours over iterations. As we do not require many candidate models of agent *j* (due to the I-DID model constraints), the fitness values converges quickly after around 100 iterations. The middle panel of Fig. 4 shows that the PSB and ACB algorithms generate new models that exhibit more diversity than GA. Given such diverse models, agent *i* is not punished when agent *j* plays actions that are not frequently seen in the interaction. The lower panel of Fig. 4 shows the average rewards that agent *i* receives when it plays with every true model of agent *j* within the candidate models. Both the PSB ad ACB algorithms achieve better performance while GA shows static rewards particularly in a short time horizon *T*=3 and 4. By examining the new models, the new PSB and ACB algorithms provide more diverse models (to be verified in Fig. 4) than GA, which helps agent *j* cope with *less-probable* behaviours of agent *j*. This is further demonstrated when the *Exact* method provides manually-made candidate models that are less random than others. Agent *i* suffers in the interaction when agent *j* executes some actions that have small probabilities in the behaviour model. Notice that the *Exact* method does not show any data for the agent *j*’s models ($m_9$-$m_{12}$ or $m_7$-$m_{9}$). This is because that the *Exact* method can’t fully cope with the scenario when agent *j*’s behaviours are not taken from the candidate set. We don’t capture the full set of data on this occasion. We further investigate this in the next section.

#### Algorithm performance when the true model of agent j is not within the candidate models

**Fig. 5:**
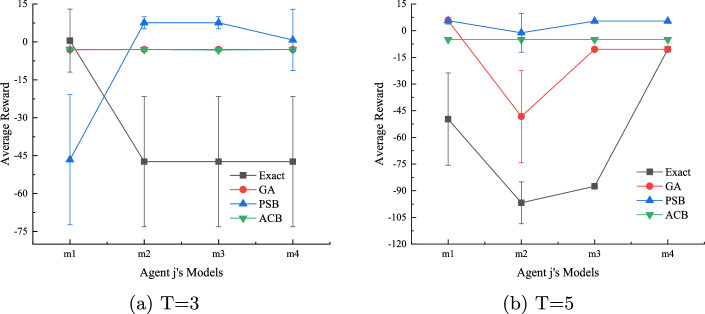
Average rewards received by agent *i* when the true model of agent *j* is not considered by agent *i*. The I-DID models used in the experiments are the same as those in Fig. 4

We consider the challenging case when the true model of agent *j* is not considered by agent *i* in the initial I-DID model. We randomly generate four models ($m_1$-$m_4$ that are not in the initial twelve candidate models). We let agent *i* use the I-DID models built in the previous experiments, and agent *j* apply the optimal policies from these initial models. As expected, all the GA, PSB and ACB algorithms outperform *Exact* in Fig. 5. The *Exact* method has little chance to capture unexpected behaviours of agent *j* while the diverse set of new models provided by other algorithms offer better chance to agent *i*. Both GA and ACB perform the same in Fig. 5 (*a*) when there is a short planning horizon of *T*=3. In general, the PSB and ACB algorithms have better performance than GA in most cases. This is particularly true for PSB.

#### Impact of parameters in the PSB and ACB algorithms

**Fig. 6:**
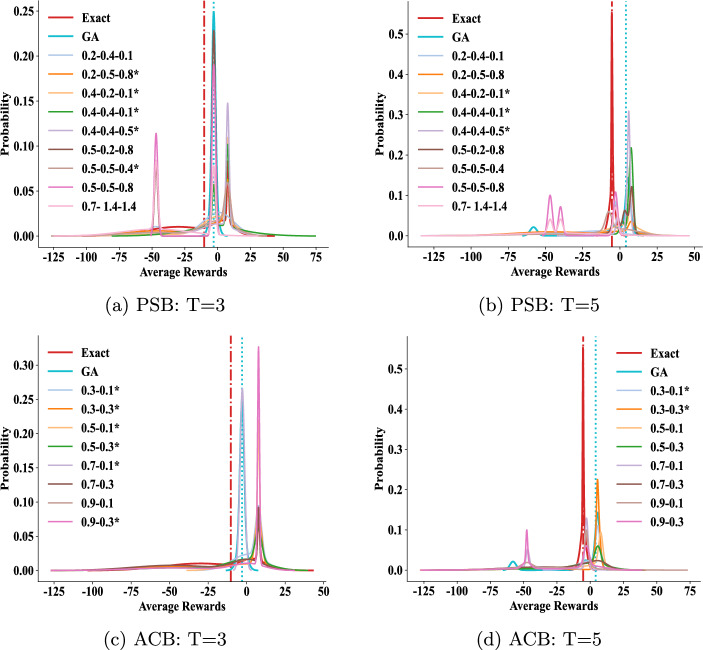
We explore parameter impacts on agent *i*’s average rewards. Figures (*a*)-(*b*) show effects of $\omega$, $c_1$, $c_2$ in PSB, while Figs. (*c*)-(*d*) show $\rho$, $\epsilon$ in ACB. See Table 1 for optimal settings. Curves with $*$ indicate better performance than Exact and GA

We further explore the impact of varying parameters in Algorithms 1 and 2. To assess the efficacy of the models derived from each parameter set, we analyze the distributions of average rewards earned by agent *i* across eight models of agent *j*, depicted in Fig. 6. In Fig. 6, the *x*-axis represents the spectrum of potential average rewards, while the *y*-axis signifies the likelihood of attaining specific rewards among the eight models. A model’s response curve nearer to the right side of the coordinate axes suggests higher reward attainment.

For reference, we plot the performance of the *Exact* and GA methods, along with the median line of their curve areas, as vertical lines (red and blue respectively in the figure). When the median line of a curve area is to the right of the benchmark lines of these two methods, we mark it with an asterisk (*) in the legend, indicating that the corresponding parameters constitute a feasible and superior set of parameters.

In our parameter sensitivity experiments, we incorporate standard PSO parameters: $\omega =0.7$, $c_1=1.4$, and $c_2=1.4$, to confirm their appropriateness for the enhanced PSO algorithm introduced here. As Eq. 4 illustrates for PSB, these parameters regulate the initial particle speed, speeds towards local and global optima, respectively. Figure 6 (*a*-*b*) doesn’t explicitly indicate the optimal parameter combination, but a well-balanced mix of these three speeds generally enhances performance across varying population sizes in PSB.

In the ACB algorithm, $\rho$ in Eq. 8 governs action retention in new behaviors, while $\epsilon$ controls greedy exploration of novel actions. Higher $\rho$ and $\epsilon$ values increase action alterations. Figure 6 (*c*-*d*) shows ACB’s stable, superior performance with increased randomness in behavior generation (especially with larger $\epsilon$), but a small $\epsilon$=0.1 in (*d*) for *T*=3. Decreasing $\rho$ with more time slices enhances ACB’s stability. Optimal parameters for PSB and ACB are in Table 1.

### The multiagent UAV problem domain

**Fig. 7:**
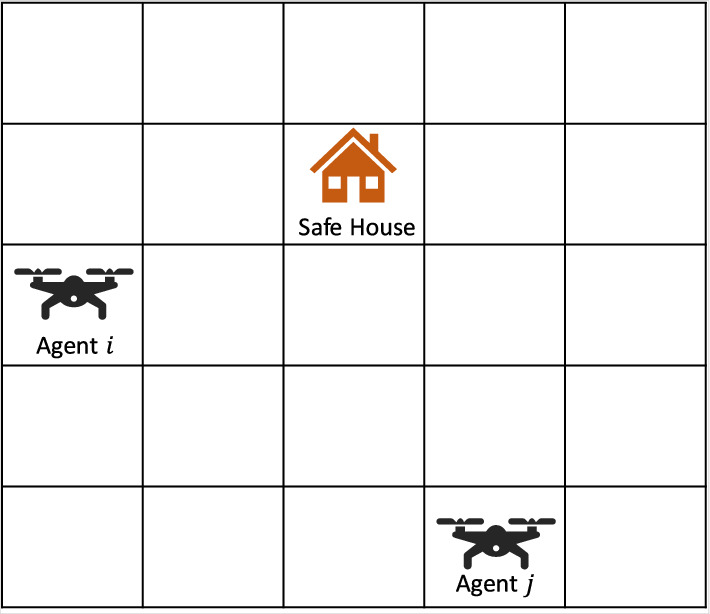
In the two-UAV problem domain, agent *i* acts as the predator, aiming to hunt down agent *j*, while the prey agent *j* flees towards the safe house to evade pursuit

The unmanned aerial vehicle (UAV) planning problem is the most complicated application in the I-DID research (Pan et al. 2023). We experiment the I-DID solution in the two-UAV version as shown in Fig. 7. In the 5x5 grid of a partially observable environment, the two UAVs do not know each other’s exact location, but are able to receive signals of their relative locations. Each of them has five actions: move up, move down, move left, move right and keep still. The predator agent *i* plans its actions over a number of steps given what it observes from the environment. The observation helps agent *i* predict what possible moves of the prey agent *j* who aims for the safe house. The number of environmental states is |*S*|=25, the observation number is $|\Omega |$=9 and the action number is |*A*|=5. The objective of this research is to explore the algorithms’ ability to handle unknown behaviors of other agents, rather than to demonstrate their scalability (which can be evaluated in the linked applications). We show the results for small values of *T*=3 and 4, which are easy to be interpreted.

**Fig. 8:**
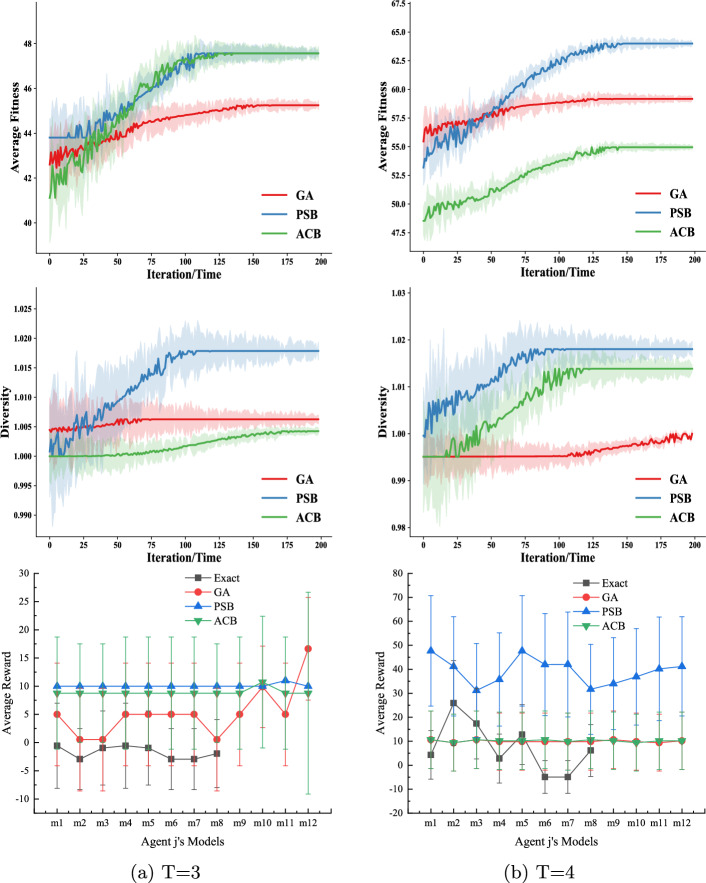
We compare four methods based on convergence speed, model diversity, and average rewards using agent *j*’s true model. Starting with 8 initial models and maintaining a population of 8, we conducted up to 200 iterations. Post-convergence, we analyzed the top 12 models for *T*=3, 4

Similar to the experiments in the two-tiger problem domain, the comparative performances between the *Exact* method and the other three evolutionary algorithms, e.g. GA, PSB and ACB, are demonstrated in Fig. 8. Both the average fitness values and diversity converge. Particularly, the PSB algorithm has the best performance among all the methods, which is clearly supported by the larger diversity of the new PSB-generated models. The *Exact* method does not perform well in all the cases of *T*=3 and 4. It demonstrates that the static behaviours do not work in the complex UAV problem domain. Optimal settings for PSB and ACB are provided in Table 1.

**Fig. 9:**
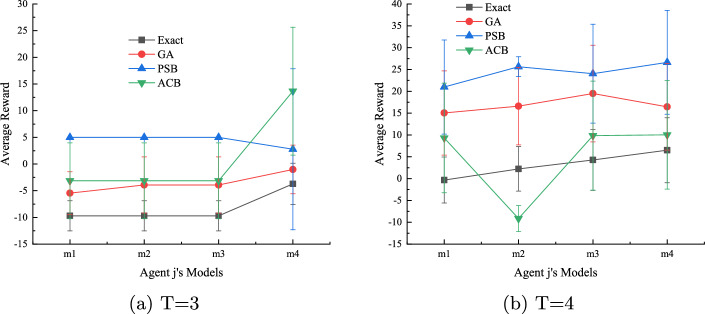
Performance of all the algorithms when the true models of agent *j* is not in the candidate model when agent *i* develops its I-DID models for $T=3$ and 4

In Fig. 9, the PSB algorithm consistently perform better in most of the cases when the true model of agent *j* hasn’t been considered by agent *i* in the I-DID models. The GA algorithm actually performs better than ACB in *T*=4, which is also consistent with the case in Fig. 8. For the UAV problem domain of a large time horizon, the GA operators do work well in generating new behaviours since the mutation operator can lead to a completely new action in a long sequence. In comparison, the *non-forgetting* paths may reduce the chance of generating new behaviours in ACB.

**Fig. 10:**
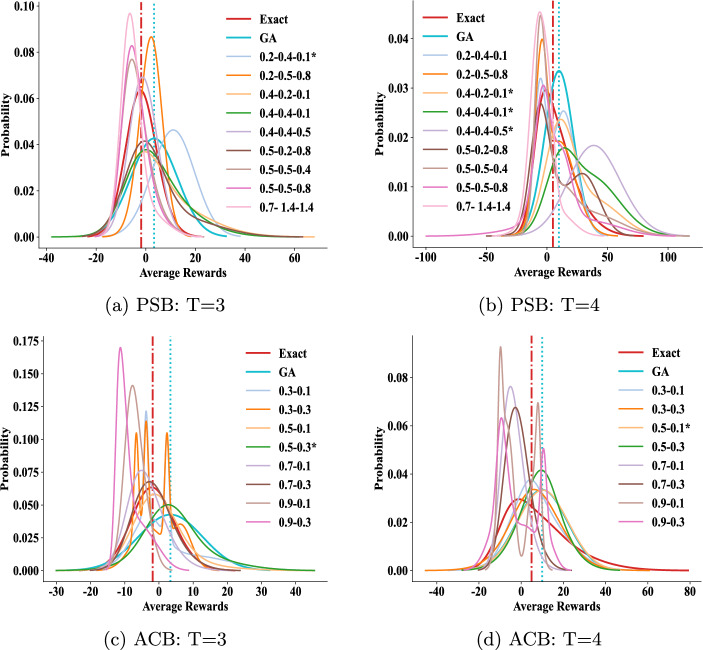
Impact of parameter values on algorithm performance. Panels (*a*-*b*) show the PSB algorithm’s performance relative to three parameters: $\omega$, $c_1$, and $c_2$. Panels (*c*-*d*) display the ACB algorithm’s performance considering two parameters: $\rho$ and $\epsilon$. Curves marked with $*$ indicate better performance than Exact and GA methods. Optimal parameter settings are in Table 1

We investigate the impact of parameter values on the performance of the PSB and ACB algorithms, as illustrated in Fig. 10. For the PSB algorithm, a higher $\omega$ value is essential to guide particles towards the global optimum as time slices progress. This indicates that emphasizing the particles’ current velocity aids in quick convergence to the optimal position. Clearly, $\omega$ significantly affects PSB’s performance. As for the ACB algorithm, $\epsilon$ is a crucial factor in determining its effectiveness. A smaller $\epsilon$ prompts ants to consider optimal actions in the current iteration more carefully, thus facilitating swift convergence to an optimal solution as time slices advance.

**Table 1:** The parameter setting in the experiments

Domain	T	PSB			ACB	
		$\omega$	$c_1$	$c_2$	$\rho$	$\varepsilon$
Tiger	3	0.4	0.4	0.5	0.5	0.1
	4	0.4	0.2	0.1	0.3	0.1
	5	0.4	0.4	0.1	0.3	0.3
UAV	3	0.2	0.4	0.1	0.5	0.3
	4	0.4	0.4	0.1	0.5	0.1

### Efficiency and statistical tests

Upon analyzing the results, it becomes evident that the PSB algorithm exhibits superior performance in terms of both behavioral diversity and reward attainment. This is attributed to PSB’s unique ability to effectively mitigate mode collapse, a common challenge in generative models, thereby enabling it to produce a wide array of potential behaviors for agent *j*. This diversity in behaviors allows agent *i* to make more informed and optimal decisions in interactive scenarios, ultimately leading to significantly higher rewards. The PSB algorithm stands out as the most effective method in this comparative analysis, due to its capacity to generate diverse and rewarding behaviors for agent *j*, which in turn enhances agent *i*’s performance.

In Table 2, we compare the running times of four algorithms for generating candidate models and solving I-DID models. Due to the many iterations needed to generate new models, PSB, ACB, and GA consume more time than *Exact*. PSB proves more efficient than GA, whereas ACB generally takes longer, needing extra iterations for a suitable candidate model set for agent *j*. Since all algorithms function off-line, efficiency is not a limiting factor at this stage. Tables 1 present optimal parameters from sensitivity analysis for PSB and ACB algorithms.

**Table 2:** Methods’ Running Times (sec) in Two Domains

Domain	T	Algorithms				Speedup	
		Exact	GA	PSB	ACB	GA/PSB	GA/ACB
Tiger	3	0.26	8.8	8.36	8.92	1.05	0.99
	4	32.41	45.1	44.47	50.31	1.01	0.90
	5	1199.41	1467.81	1277.7	1346.49	1.15	1.09
UAV	3	8.16	27	29.43	28.62	0.92	0.94
	4	406.57	637.18	604.17	1026.67	1.05	0.62

We conducted paired *t*-tests to assess algorithm reliability across two domains, comparing each pair for significant performance differences. The *p* values (significance level: 0.05) are in Table 3. In Table 3, $H_0$ and $H_1$ denote null and alternative hypotheses. Results indicate PSB or ACB outperforms others, regardless of other agents’ true behavior within a subject agent’s behavioral space. In summary, comprehensive experiments show that PSB and ACB outperform GA and Exact in most cases. PSB’s consistent performance may be due to PSO’s balance of coherent and individual behaviors, while ACO leads to monotonic paths. Finding optimal parameters remains crucial, depending on problem domains and complexity. Additionally, SI-based I-DID solutions model unexpected agent behaviors, unachievable by classical AI. Despite SI’s randomness, experimental results show stable average rewards for the subject agent, consistent with our theoretical analysis.

**Table 3:** t-Test Results for Model Comparison

*j*’s model	$H_0$	$H_1$	Tiger			UAV	
			T3	T4	T5	T3	T4
True	Exact>GA	GA>Exact	0.0045	0.047	0.0056	0.0213	0.043
	Exact>PSB	PSB>Exact	0.0034	0.0009	0.0023	0.0045	0.0011
	Exact>ACB	ACB>Exact	0.0012	0.033	0.047	0.0143	0.041
	GA>PSB	PSB>GA	0.0471	0.0015	0.034	0.0034	0.0032
	GA>ACB	ACB>GA	0.0021	0.043	0.062	0.0451	0.054
	PSB>ACB	ACB>PSB	0.0015	0.0016	0.071	0.0012	0.0012
Not True	Exact>GA	GA>Exact	0.001	-	0	0.0034	0.0023
	Exact>PSB	PSB>Exact	0.0018	-	0.0024	0.0021	0.0011
	Exact>ACB	ACB>Exact	0.001	-	0.0019	0.0035	0.021
	GA>PSB	PSB>GA	0.0034	-	0	0.0019	0.0061
	GA>ACB	ACB>GA	0	-	0.0234	0.043	0.0019
	PSB>ACB	ACB>PSB	0.0175	-	0	0.0053	0.0013

### Extensions, discussions and limitations

We make a further step to explore the PSB/ACB enabled I-DID solutions in more complex multiagent settings and discuss their limitations.

#### More Agents

The SI-based algorithms have shown the potential of dealing with unexpected behaviours of other agents in I-DIDs and contributes to the traditional AI planning techniques in multiagent systems. The benefit arises from heuristic operators that evolve potential agents’ behaviours in the PSB/ACB algorithms. The operations indeed require a large amount of computational times to find rationally sound behaviors and become more complex when more agents are considered in problem domains.

We let agent *i* interact with 3, 5 and 7 agents in both multiagent tiger and UAV problem domains. In the tiger problem domain, agent *i* receives the money only if all of them open the door in a collaborative way; otherwise, it is to be eaten by the tiger. Hence, it becomes more difficult when agent *i* needs to reason with behaviours of other agents. In the UAV problem domain, we extend the grid environment into 11X11 states, and the predator agent *i* gets awarded when it either captures another agent or blocks their way to the safe house. We use either GA, PSB or ACB to generate potential behaviours for another agent. The *Exact* method can’t seriously cope with unexpected behaviours of other agents particularly when more agents are involved in the interaction. Since conducting the experiments of I-DIDs with other planning horizons requires extra times, we only show the experimental results of *T*=5 in Table 4.

**Table 4:** Average rewards achieved by agent *i* with more agents over 50 interactions when they play for *T*=5. The experimental setting is the same as that of the previous experiments

Domain	Methods	3 Agents		5 Agents		7 Agents	
		Mean	Std	Mean	Std	Mean	Std
Tiger	GA	$-$3.13	0.43	0.43	1.31	$-$0.32	1.56
	PSB	3.56	0.12	3.34	1.2	2.35	0.34
	ACB	1.22	0.32	2.23	1.09	0.48	1.1
UAV	GA	19.1	3.45	23.11	2.1	18.76	3.45
	PSB	34.12	2.13	38.42	1.9	36.17	2.4
	ACB	25.89	1.99	29.78	2.41	20.1	3.12

The PSB algorithm performs consistently better than the GA and ACB algorithms for all the cases of many agents. By examining the running times in Table 5, all the algorithms need to consume around hours (especially in the UAV problem domains) to generate the candidate set of other agents’ behaviours. Although the computations are executed offline, the process is rather tedious and may become unsustainable in more complex cases. As generating potential behaviours of every agent is independent, it deserves the investigation of executing the behavioural generation in a distributed way, which may significantly reduces the computational times.

**Table 5:** Times (in minutes) for the comparative methods for many agents

Domain	Methods	3 Agents	5 Agents	7 Agents
Tiger	GA	80.12	140.67	178.22
	PSB	40.45	56.23	134.12
	ACB	49.12	89.9	160.9
UAV	GA	112.37	274.38	312.97
	PSB	70.98	189.12	276.73
	ACB	87.12	203.45	309.19

#### Mixed cooperative and competitive settings

We explore the application of new I-DID solutions in a complex problem domain of organization management that involves a mix of cooperation and competition among employees (He et al. 2024). Following the problem specification (He et al. 2024) in this domain, we have one manager and and six possible employees. The employees need to work together to increase the organization’s revenue while competing for a maximal share of the bonus pool. They have four possible actions: working for self-interest (self), trading off the organization and personal benefits (balance), working solely for the organization (group), and even resigning from the group (resign). The manager have two extra actions of hiring new employees and firing incompetent ones. The organization’s financial health status is categorized into five - from the low to very high levels, and can’t be observed directly. The manager and employees can inferred the states through the observation of the organization’s orders - the three levels of orders as meager, several, and many. They agents receive their rewards depending on the organization financial states and their joint actions.

We let the manager be the subject agent *i* who models and interacts with the other six agents *j*. Since the PSB/ACB algorithms are used to generate potential behaviours for every other agents, we can’t show various plots of the diversity and fitness change over the iterations, but report the average rewards for running 50 interactions over *T*=4 and $T=6$ in Table 6. We let the mange stick to the specific I-DID model built through the algorithms and interact with their employees who selects their individual models in a random basis. Both the PCB and ACB algorithms still show better performance than the GA algorithm. This may stem from the benefit of PCB/ACB on generating better collective behaviours while still preserving the behavioural diversity. The benefit is more visible when more sophisticated relations exist in a problem domain. For example, the employees may form a certain degree of cooperation for a short period and compete with each others in the remaining times. Meanwhile, the PSB algorithm continues to exhibit efficiency for generating the potential behaviours in the I-DID model development times. However, we shall point out that the I-DID model can’t run the interactions for many planning horizons due to the scalability issue that stems from the explicitly structural, explainable decision model. It is more suitable to be used when the organization requires short-time, yet transparent management.

**Table 6:** Average rewards achieved by agent *i* with more agents over 50 interactions when they play for *T* =4 and 6. The experimental setting is the same as that of the previous experiments

Domain	Methods	Average Rewards				Times (minutes)	
		T=4		T=6		T=4	T=6
		Mean	Std	Mean	Std		
Org	GA	0.714	0.022	2.327	0.087	23.8	34.76
	PSB	2.45	0.013	4.786	0.045	15.46	23.34
	ACB	1.98	0.078	4.121	0.063	18.3	28.98

#### Limitations

The parameter adjustment poses the difficulty on implementing SI-based I-DID as we shown in the sensitivity analysis. This is unavoidable since the algorithms inherit the nature of the randomness from evolutionary algorithms. Although we have provided operational guidelines (in Fig. 6 and Fig. 10) on adjusting the parameters in the aforementioned problems, a more systematic development of parameter analysis could be explored (Gomes Pereira de Lacerda et al. 2021). Some visualization techniques, e.g. showing the algorithm performance against a combination of parameter values, would help the value selection. The linked I-DID toolkit can facilitate such analysis.

It is not the prime goal of testing the scalability of SI-based I-DID solutions in terms of planning horizons (*T*) in this article. As the SI techniques are for the purpose of generating new behaviours, they will maintain the same scalability as other I-DID solutions. According to the experiments of more complex problem domains, the PSB/ACB algorithms can’t fully show the potential to exploit the sophisticated relations among multiple agents in the problem domains. We may explore the agents’ local structure in trust-based communities (Fotia et al. 2017) and further examine the algorithm’s utility.

The PSB/ACB time-consuming iteration will definitely increase the overhead of solving I-DIDs for larger planning horizons. Other approaches, e.g. deep reinforcement learning or other generative methods (He et al. 2024), shall be explored in parallel to the SI-based I-DID solutions. One relevant note that we may use SI-based techniques to improve the I-DID scalability since a complex policy tree could be encoded and manipulated efficiently through the evolutionary operators.

## Conclusion and future work

By considering the strengths of collective behaviours in swarm intelligence, we adapt PSO and ACO algorithms to solve the new behavioural optimization in I-DIDs. The new methods (PSB/ACB) have the opportunity of capturing unexpected behaviours of other agents, which is rarely exploited in the recent AI research. We theoretically analyze the correctness and reliability of the new methods. According to the empirical results, we observe that PSB has the strength of generating individual behaviours associated with different beliefs while exploiting collective behaviours in the generation.

One potential complexity would be in evaluating individual behaviours in every iteration of PSB/ACB. We will continue the experiments in more problem domains while exploring different ways in the behavioural evaluation. The linked I-DID toolkit can be used to run any experiments including the sensitivity analysis of the experimental parameters, the full results can’t be included in the limited article space. We will develop an efficient and intuitive design of the parameter analysis in the implementation, which will facilitate the value selection for the algorithmic parameters in a smart way. In addition, our method is an improvement and complement to the research on dynamic response strategy optimization in multiagent decision making. The issue of how the subject agent can timely update its own decision in real time through the behaviour model of other agents is still complex.

## Supplementary Information

Below is the link to the electronic supplementary material.

Supplementary file 1 (pdf 608 KB)

## Data Availability

All the data and implementation could be accessed through the link: https://github.com/lamingic/Ev-IDID and https://github.com/lamingic/SI-IDID.

## Footnotes

1 https://github.com/lamingic/SI-IDID
