# Supplementary material for: Improved decisions for unknown behaviours in interactive dynamic influence diagrams
Source: Artif Intell Rev. 2025 Aug 30;58(11):361. doi: 10.1007/s10462-025-11355-y (PMC12398443; doi:10.1007/s10462-025-11355-y)
Supplement: Supplementary file 1 — (pdf 608 KB) [file 10462_2025_11355_MOESM1_ESM.pdf]

Supplemental Material for "Improved Response Strategies for Unknown Behaviours in Multiagent Interaction"

Yinghui Pan, Mengen Zhou, Biyang Ma, Yifeng Zeng,
Yew-soon Ong, Guoquan Liu

In this Supplementary Materials, we detail the frameworks of PSB and ACB, PSB operators, and shared functionalities. All the implementations are included in the I-DID toolkit [1] .

A: PSB and ACB Implementation

We introduce PSB and ACB frameworks in Figs. 1 and 2, respectively, and elaborate on PSB operators and their shared functionalities with ACB.

A.1: [The framework of PSB and ACB]

Fig. 1 illustrates our PSO-enabled Behavior (PSB) framework. Initially, known policy trees are represented as particles $\sigma = (p, v)$, forming the population pop (①). Each particle's position p is constructed from the sequential actions in the policy tree, while its velocity v is initialized as an empty set. Fitness $F(\sigma)$ is calculated for each particle (②) using the GeNIe toolkit, enabling the identification of local best $\bar{\sigma}$ and global best σ^* particles (③). Over N iterations, particles update their positions and velocities (④-⑤) based on the positions of the global and local optimal particles, using defined operators. Fitness is recalculated (⑥), and the best particles are updated (⑦). Each particle moves towards either σ^* or $\bar{\sigma}$ (⑧). Ultimately, particles are transformed back into policy trees and decoded into behaviors via the $transform$ operator (⑨). The top-K behaviors, based on fitness, are selected (⑩). Fig 1 provides an example of this framework applied to a dataset $\mathcal{D}^{T=3}$, demonstrating how behaviors, initialized with diverse beliefs, are optimized over iterations to maximize the diversity of output behaviors.

Fig. 2 introduces our Ant Colony Optimization-enabled Behavior (ACB) framework. The framework initializes ant populations and pheromone tables (①-②). It calculates the pheromones for each ant using the GeNIe toolkit, which indicates the expected reward for actions in different positions. Pheromones are updated based on

[1] https://github.com/lamingic/SI-IDID

the actions taken by the ants (③-④). Ants select their next actions in an epsilon-greedy manner, influenced by the pheromone levels (⑤). After N iterations (⑥), the ant population is converted back into policy trees, and the top-K behaviors are selected based on their fitness (⑦-⑧). Fig 2 showcases an example of this framework applied to a dataset $\mathcal{D}^{T=3}$, illustrating how behaviors, initialized with a variety of beliefs, are optimized over iterations to enhance the diversity of the output behaviors.
A.2: [PSB Operators]

We introduce four PSB operators:

- *Minus* ($\ominus$): Computes particle velocity from positions.
- *Plus* ($\oplus$): Calculates new particle positions.
- *Times* ($\otimes$): Scales velocity v_1 by scalar ω.
- *Merging* ($\uplus$): Combines velocities v_1 and v_2.

A.3: [The Common Operators in PSB and ACB]. Both PSB and ACB algorithms utilize four fundamental operators detailed in Alg. 2. The operators in PSB and ACB enable transformations between policy trees and particles/ants.

- Formalise: Generates an action sequence from a policy tree.
- Transform: Converts action sequences to policy trees.
- Fitness: Evaluates particle sequences using GeNIe decision tool [2].
- Evaluate: Calculates pheromone for ant policies.

[2]https://www.bayesfusion.com

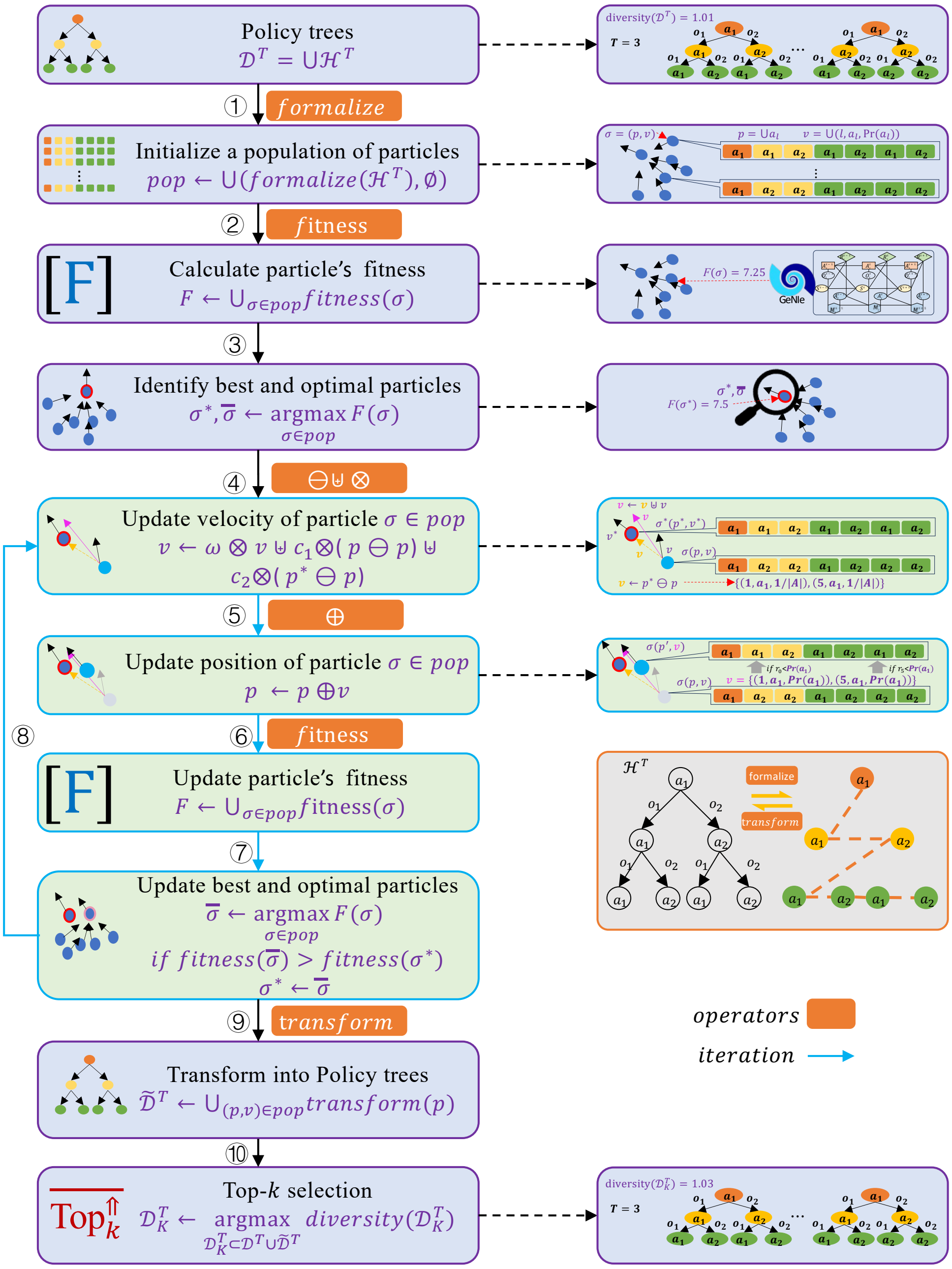

Fig. 1: The PSO-enabled behavior generation framework, exemplified with a dataset $\mathcal{D}^{T=3}$, comprises main components: initialization of particles from known policy trees, updates of particles' positions and velocities based on global and local optima, and selection of top-K behaviors based on fitness.

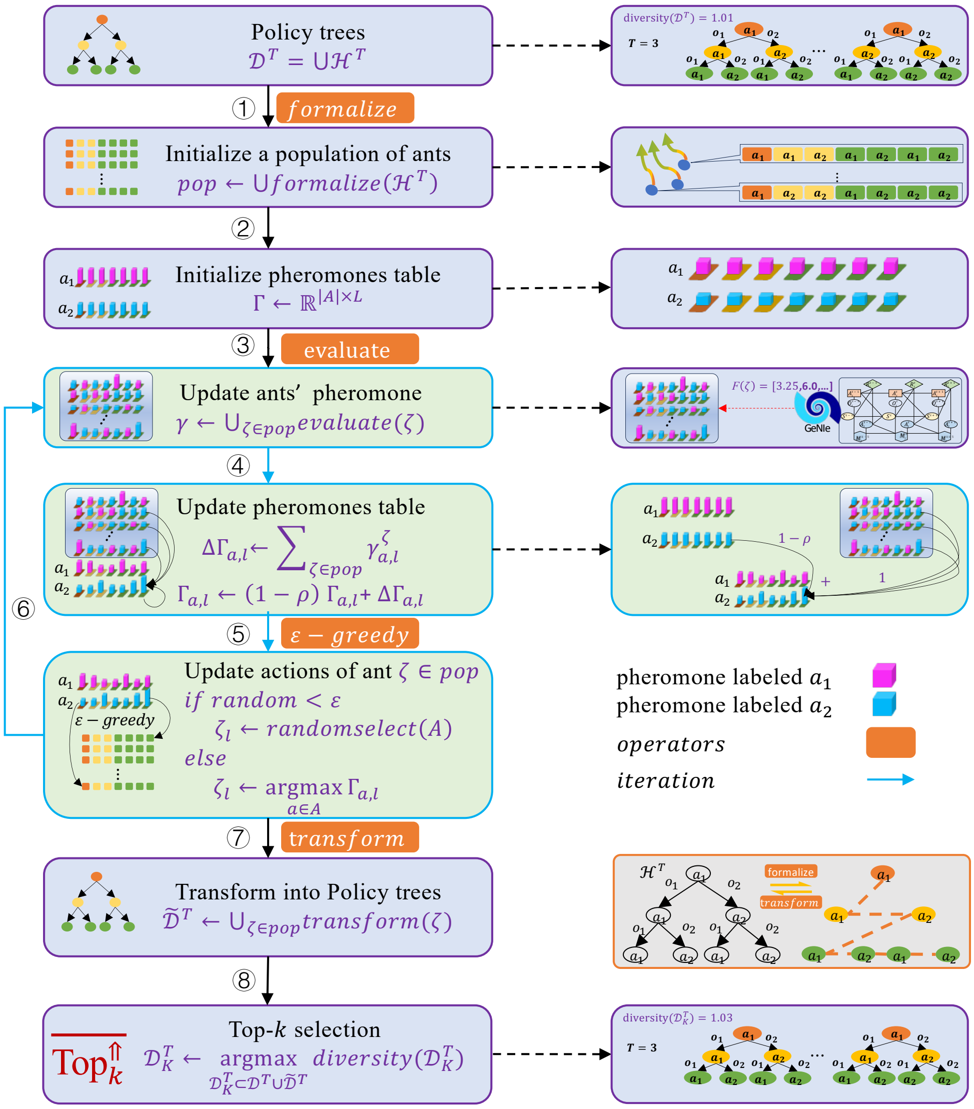

Fig. 2: The ACB algorithm applied to a dataset $\mathcal{D}^{T=3}$, where new behaviors are generated by iteratively updating the pheromone table and maintaining an ant population. Each ant performs action sequences, which are updated based on the selection of new actions, enhancing the diversity of the output behaviors.

Algorithm 1: The Four Operators in PSB

```
Function ⊖( p1, p2 )    ◁ v ← p1 ⊖ p2:
    v ← ∅
    for l ∈ {1, 2, ··· |p1|} do
        if p1[l] ≠ p2[l] then
            v ← v ⋃(l, p1[l], 1/|A|)
        end
    end
return v
Function ⊕( p1, v )    ◁ p ← p1 ⊕ v:
    p ← p1
    for (l, a, P) ∈ v do
        if random() > P then
            a ← randomselect(A)
        end
        p[l] ← a
    end
return p
Function ⊗( v1, ω )    ◁ v ← ω ⊗ v1:
    v ← ∅
    for (l, a, P) ∈ v1 do
        v ← v ⋃(l, a, min(ω ∗ P, 1))
    end
return v
Function ⊎( v1, v2 )    ◁ v ← v1 ⊎ v2:
    v ← ∅
    for (l, a, P) ∈ v1 do
        for (l′, a′, P′) ∈ v2 do
            if l == l′ & a == a′ then
                v ← v ⋃(l′, a′, min(P + P′, 1))
            end
            else
                v ← v ⋃(l, a, P)
                v ← v ⋃(l′, a′, P′)
            end
        end
    end
return v
```

Algorithm 2: The operators in PSB and ACB

Function $formalise(\ \mathcal{H}^T)$**:**

$\triangleleft$ *generate action sequence of tree* $\mathcal{H}^T$

$p \leftarrow \{a_l | a_l \leftarrow 0, \forall l \in \{1, 2, \cdots \frac{|\Omega|^T - 1}{|\Omega| - 1}\}\}$

for $(h^T, i) \in \mathcal{H}^T$ **do**

$\{a_1, o_2, \cdots, o_T, a_T\} \leftarrow h^T$

for $t \in \{1, 2, \cdots T\}$ **do**

$j \leftarrow \frac{|\Omega|^{(t-1)} - 1}{|\Omega| - 1} + \lfloor \frac{i-1}{|\Omega|^{(t-1)}} \rfloor + 1$

$p[j] \leftarrow a_t$

end

end

return p

Function $transform(p)$**:**

$\triangleleft$ *generate policy tree from action sequence* p

$\mathcal{H}^T \leftarrow \bigcup h^T$

for $(h^T, i) \in \mathcal{H}^T$ **do**

$\{a_1, o_2, \cdots, o_T, a_T\} \leftarrow h^T$

for $t \in \{1, 2, \cdots T\}$ **do**

$j \leftarrow \frac{|\Omega|^{(t-1)} - 1}{|\Omega| - 1} + \lfloor \frac{i-1}{|\Omega|^{(t-1)}} \rfloor + 1$

$a_t \leftarrow p[j]$

if $t \neq T$ **then**

$k \leftarrow \lfloor \frac{(i-1)|\Omega|^t}{|\Omega|^{(T-1)}} \rfloor + 1$

$o_{t+1} \leftarrow o^k$

end

end

$h^T \leftarrow \{a_1, o_2, \cdots, o_T, a_T\}$

end

return $\mathcal{H}^T$

Function $fitness(\sigma)$**:**

$\triangleleft$ *evaluate the fitness of action sequence* σ

$\mathcal{H}^T \leftarrow transform(\sigma)$

for $s_i \in S$ **do**

$V_{\mathcal{H}^T}(s_i) \leftarrow R(s, a(\mathcal{H}^T)) + \lambda \sum_{\forall s' \in S} Pr_{a(\mathcal{H}^T)}(s'|s)[\sum_{\forall o \in O} Pr_{a(\mathcal{H}^T)}(o|s') V_{o(\mathcal{H}^T)}(s')]$

end

$\alpha_{\mathcal{H}^T} \leftarrow [V_{\mathcal{H}^T}(s_1), V_{\mathcal{H}^T}(s_2), \cdots, V_{\mathcal{H}^T}(s_{|S|})]$

$f \leftarrow \sum_{\forall s \in S} b^0(s) \alpha_{\mathcal{H}^T}(s)$

return f

Function $evaluate(\zeta)$**:**

$\triangleleft$ *evaluate the expected reward of action sequence* σ

$\mathcal{H}^T \leftarrow transform(\zeta)$

$ER \leftarrow \mathbb{R}^{|A| \times \frac{|\Omega|^T - 1}{|\Omega| - 1}}$

for $(h^T, i) \in \mathcal{H}^T$ **do**

$\{a_1, o_2, \cdots, o_T, a_T\} \leftarrow h^T$

for $t \in \{1, 2, \cdots T\}$ **do**

$l \leftarrow \frac{|\Omega|^{(t-1)} - 1}{|\Omega| - 1} + \lfloor \frac{i}{|\Omega|^{(t-1)}} \rfloor$

for $s \in S$ **do**

$b^t(s) \leftarrow \frac{Pr(o_l | s, a_l \sum_{s' \in S} Pr(s|s', a_l) Pr(s'|b^{t-1})}{\sum_{s' s'' \in S} Pr(o_l|s'', a_l) Pr(s''|s', a_l) Pr(s'|b^{t-1})}$

end

$ER(a_l, l) \leftarrow \sum_{s \in S} b^t(s) R(s, a_l)$

end

end

return ER
